# Editorial: Infection-mediated inflammation that promotes cancer initiation and/or progression

**DOI:** 10.3389/fmed.2025.1564726

**Published:** 2025-02-11

**Authors:** Ibrahim M. Sayed, Soumita Das

**Affiliations:** ^1^Department of Biomedical and Nutritional Sciences, University of Massachusetts Lowell, Lowell, MA, United States; ^2^Department of Medical Microbiology and Immunology, Faculty of Medicine, Assiut University, Assiut, Egypt

**Keywords:** microbe, cancer, inflammation, cross-talk between cells, infection, signaling pathways

Microbial infection mediates the initiation and progression of cancer through several strategies. These strategies include the stimulation of host inflammatory responses (Infection-mediated inflammation), upregulation of oxidative DNA/RNA damage and production of reactive oxygen species (ROS), suppression of host repair mechanisms, and uncontrolled proliferation of host cells (1, 2). There are several bacteria that mediate cancer programs via infection-mediated inflammation such as *Helicobacter pylori* (*H. pylori*), *Fusobacterium nucleatum, Enterotoxigenic B. fragilis, Clostridium difficile, and Enterococcus faecalis* (1, 3). *H. pylori* is a gram-negative bacilli that cause gastric cancer, colon cancer, and extra-intestinal cancers (1). The pathogenesis of *H. pylori* includes the following pathways; upregulation of inflammatory signaling pathway via NF-κB stimulation, increasing DNA/RNA oxidative damage and inhibiting the host repair pathways, inducing the epithelial cell proliferation and suppression of the tumor suppressor protein P53 (1). In this Research Topic, two studies discussed the pathogenesis of *H. pylori* infection. Elbehiry et al. described the role of *H. pylori* virulence factors in bacterial pathogenesis including outer membrane proteins (OMPs), enzymes (such as catalase and urease), and toxins [such as vacuolating cytotoxin gene (*vacA*) and cytotoxin-associated gene A (*cagA*)]. Bawali et al. reported the role of extracellular vesicle connection in driving inflammation and gastrointestinal tract cancers. The outer membrane vesicles (OMVs) of *H. pylori* and host-cell-derived extracellular vesicles (EVs) mediate the transport of carcinogenic cytotoxin of *H. pylori*, the cytotoxin-associated gene A (*CagA*). *CagA* diminishes the host immune response, induces inflammation in the gastric mucosa by stimulating IL-8 and nuclear factor-κB (NF-κB), and upregulates the reactive oxygen species (ROS) generation. EVs released by infected cells contain *CagA*, reach systemic circulation, and deliver the oncogenic factors to the distal parts of the body. The OMVs of *H. pylori* induce extra gastric disease such as hepatic fibrosis by affecting the exosomes in liver cells and stimulating hepatic satellite cells. Also, the fusion of *H. pylori* OMVs with other microbial OMVs, which is pH-dependent, can be an oncogenic factor for extra gastric cancers. The authors conclude that the advancement of EV research and bioengineering and OMV-OMV fusion could be efficient therapeutic targets (Bawali et al.). Regarding the development of effective vaccine against *H. pylori* infection, Elbehiry et al. described several vaccine approaches which are developed for *H. pylori* including inactivated whole-cell vaccines, genetically modified protein-based subunit vaccines, *cagA* antigen as a vaccine candidate, multiepitope DNA vaccines, and vector (carrier) vaccines. However, the efficacy of several vaccine types is unknown and most of the research findings are preliminary. The OMP- vaccine is safer and more effective in reducing antibiotic resistance. Toxin vaccines have some degree of safety but challenge the selection process. Current clinical trials assess recombinant vaccines incorporating various antigens such as the *cagA, vacA, ure, babA, sabA, oipA*, and porin proteins. The ideal vaccine against *H. pylori* should involve the immune-suppressing mechanisms and the selection of effective antigens and adjuvants. Besides, testing the potential candidate vaccines should be performed in a suitable animal model before human clinical trials.

Immune cells play critical roles in the development of oral cancer. Lymphocytes and macrophages are the main players in anti-tumor immunity. Shifting from CD8^+^ T cells into CD4^+^ T cells and cytokine profile have been linked to cancer progression (4). The cytokines that enhance tumor microenvironment include PGE-2, TGF-β and IL-10 (4). Chronic infection and crosstalk between the immune cells and neural cells could promote oral cancers. In this Research Topic, D'Silva and Pandiyan reviewed neuroimmune cell interactions and chronic infections in oral cancers, including the immunomodulatory programs that affect cancer progression. The authors showed that the depletion of CD8^+^ T cells and increased FOXP3^+^ regulatory T-cells affect immune homeostasis, chronic infections, and tumor progression. During inflammation and tissue injury, nerves affect immune regulation through the release of neurotransmitter neuropeptides such as calcitonin gene-related peptide and substance P, also glial cells produce prostaglandin E2. During tumor progression, several neurotrophic factors play a role in the interaction between neural cells and immune cells, such as Neuregulins, Amphiregulin (AREG), and Schwann cell-derived AREG. The previous agents affect nerve-tumor-immune cell crosstalk through activation of signaling pathways in leukocytes such as the EGFR, the ERK/MAPK, the PI3K/AKT, mTOR signaling pathway, and the JAK-signal transducer and STAT pathway. The authors concluded that cellular dysregulation related to microbiome dysbiosis, infections, and chronic inflammation affect the development of oral cancers (D'Silva and Pandiyan).

Not only bacteria, but also viruses play a role in cancer development. Viruses such as Hepatitis B virus, Hepatitis C virus, Epstein Barr virus, and Human papillomavirus are reported with several human cancers such as liver cancer, Burkitt's lymphoma, and cervical cancer. There are several strategies of virus-induced cancers such as activation of oncogenes, downregulation of tumor suppressor proteins, alteration of cellular signaling and immune response, promotion of inflammatory responses, mediating DNA damage, and inducing host cell proliferation (5). In this Research Topic, Javadi et al. assessed the correlation between human papillomavirus (HPV) infection and colorectal cancer. In a cross-section study including 40 CRC biopsies, 26 samples were positive for HPV DNA. HPV was also detected in the urine of HPV-positive patients (14/26, 53.8%). Genotyping analysis revealed that HPV16 was the most common subtype in CRC biopsies and urine samples, with no significant difference between genders. HPV45 and HPV83 were detected in CRC biopsies of male and female patients respectively. HPV31 and HPV56 were also detected in the urine of male and female patients respectively. The authors also assessed HPV16 E6 and E7 oncoproteins in exosomes from serum samples, suggesting the potential of a non-invasive method in HPV diagnosis. The authors concluded the role of HPV infection in CRC development and the impact of HPV DNA screening in CRC tissues on patients' survival (Javadi et al.).

Infections can cause death among leukemic patients due to weakened immune systems. In this Research Topic, Marvin-Peek et al. studied the prevalence of Nontuberculous mycobacteria (NTM) infections in patients with leukemia (*n* = 29,743) admitted to the University of Texas MD Anderson Cancer Center in Houston from 2016 to 2023. The identification of NTM was confirmed by acid-fast bacilli (AFB) cultures and 16S ribosomal DNA sequencing. There were six cases of NTM infection, and two patients had passed away. Although the infection rate of NTM is very low (0.02%), the death rate was high. It is crucial to consider NTM in leukemia patients with early AFB cultures and prescribe the appropriate therapies, especially in case of disseminated infection including neutropenic fevers, abnormal pulmonary nodules, or unusual skin lesions.

In conclusion: Bacterial and viral infections contribute to cancer development through different approaches including inflammation, DNA damage, and induction of cancer signaling pathways. Understanding the infection-mediated cancer programs will help in the development of effective therapeutic interventions and/or preventive vaccine approaches.
